# *Echinacea purpurea *and osteopathic manipulative treatment in children with recurrent otitis media: a randomized controlled trial

**DOI:** 10.1186/1472-6882-8-56

**Published:** 2008-10-02

**Authors:** Richard A Wahl, Michael B Aldous, Katherine A Worden, Kathryn L Grant

**Affiliations:** 1 Department of Pediatrics, University of Arizona, Tucson, Arizona, USA; 2 Department of Pediatrics, Saltzer Medical Group, Nampa, Idaho, USA; 3 Department of Osteopathic Manipulative Medicine, Midwestern University, Glendale, Arizona, USA; 4 Department of Pharmacology, University of Arizona, Tucson, Arizona, USA

## Abstract

**Background:**

Recurrent otitis media is a common problem in young children. Echinacea and osteopathic manipulative treatment have been proposed as preventive measures, but have been inadequately studied. This study was designed to assess the efficacy of *Echinacea purpurea *and/or osteopathic manipulative treatment (OMT) for prevention of acute otitis media in otitis-prone children.

**Methods:**

A randomized, placebo-controlled, two-by-two factorial trial with 6-month follow-up, conducted 1999 – 2002 in Tucson, Arizona. Patients were aged 12–60 months with recurrent otitis media, defined as three or more separate episodes of acute otitis media within six months, or at least four episodes in one year. Ninety children (44% white non-Hispanic, 39% Hispanic, 57% male) were enrolled, of which 84 had follow-up for at least 3 months. Children were randomly assigned to one of four protocol groups: double placebo, echinacea plus sham OMT, true OMT (including cranial manipulation) plus placebo echinacea, or true echinacea plus OMT. An alcohol extract of *Echinacea purpurea *roots and seeds (or placebo) was administered for 10 days at the first sign of each common cold. Five OMT visits (or sham treatments) were offered over 3 months.

**Results:**

No interaction was found between echinacea and OMT. Echinacea was associated with a borderline increased risk of having at least one episode of acute otitis media during 6-month follow-up compared to placebo (65% versus 41%; relative risk, 1.59, 95% CI 1.04, 2.42). OMT did not significantly affect risk compared to sham (44% versus 61%; relative risk, 0.72, 95% CI 0.48, 1.10).

**Conclusion:**

In otitis-prone young children, treating colds with this form of echinacea does not decrease the risk of acute otitis media, and may in fact increase risk. A regimen of up to five osteopathic manipulative treatments does not significantly decrease the risk of acute otitis media.

**Trial registration:**

ClinicalTrials.gov Identifier: NCT00010465

## Background

By age 3 years, most children (50–84%) experience at least one episode of acute otitis media (AOM). Recurrent otitis media is also common; 10% to 19% of children suffer 3 or more episodes during the first year alone [1]. In children with recurrent otitis media, conventional approaches to decreasing the risk of further episodes include prophylactic antibiotic therapy and surgical insertion of tympanostomy tubes to prevent accumulation of middle ear effusion. The effectiveness of these approaches is limited and controversial [2,3].

Complementary and alternative medicine (CAM) use in pediatric patients remains relatively common. One survey of parents at an urban pediatric emergency department reported an overall incidence of CAM use of 15%. Herbal remedies were used by 40% of those families that reported use of CAM modalities[4]. CAM approaches are often used for treatment of recurrent otitis media. A survey of pediatric clinic patients in Quebec revealed that ear, nose, and throat problems accounted for 24% of child visits to alternative medicine practitioners [5], and a survey of parents at a British pediatric otolaryngology clinic found that 29% reported CAM use, with echinacea and manipulative treatments frequently cited [6].

A recent report suggested a benefit of osteopathic manipulative treatment (OMT) in reducing episodes of AOM in otitis-prone children [7]. The osteopathic concept of recurrent otitis media postulates that predisposing structural dysfunction contributes to Eustachian tube dysfunction and is amenable to treatment with OMT, particularly in the cranial region [8-11].

Echinacea is an herb commonly used for treatment of upper respiratory tract infections. In vitro studies suggest a stimulatory effect of *Echinacea purpurea *preparations on various components of the immune system [12-14]. Several studies demonstrate improvement in the severity and duration of the common cold in adults treated with echinacea products [15]. Others, including the most rigorous study in children, show no benefit [16,17]. Because most episodes of AOM follow upper respiratory tract infections, it is plausible that treatment of such infections with echinacea might decrease the risk of subsequent AOM.

We conducted a randomized, double-blind, placebo controlled trial between 1999 – 2002 to assess the efficacy of echinacea treatment (at the time of upper respiratory infections) and OMT for the prevention of AOM in children with recurrent otitis media.

## Methods

### Definitions

No generally accepted diagnostic definition for AOM existed at the time of our study[18]. We defined AOM as an acute illness with at least one symptom (fever, rhinorrhea, cough, otalgia, irritability, lethargy, anorexia, vomiting, or diarrhea) and at least two otoscopic findings of middle-ear inflammation (injection, opacity, fullness, or impaired mobility) or purulent otorrhea. This definition is consistent with the later 2004 American Academy of Pediatrics guidelines on otitis media[19]. Recurrent otitis media was defined as at least 3 separate episodes of AOM within a 6-month period, or 4 episodes in one year. In order for episodes to be considered separate, the protocol required otoscopic documentation of resolution of one episode, or at least two weeks without symptoms following completion of antibiotic therapy, prior to onset of the subsequent episode.

### Subjects

Children aged 12–60 months with recurrent otitis media (as documented in their medical records) were recruited from the Arizona Health Sciences Center Pediatric Clinic and from private pediatric offices in Tucson, Arizona. Children were excluded for unwillingness to participate, congenital malformations of the ears, nose, or throat (e.g., cleft palate), known or suspected allergy to echinacea, immune deficiency including HIV infection, or tuberculosis. Children taking prophylactic antibiotics or who had tympanostomy tubes in place were excluded from enrollment. The Arizona Health Sciences Center Pediatric Clinic serves an urban population, of which approximately 75% are covered by Medicaid.

### Randomization

Randomization was done in blocks of eight using a random number table by a clinical pharmacist (KLG) having no contact with participants. Children were randomly assigned to one of four groups in a two-by-two factorial design. The first group received placebo extract orally and sham manipulation. The second group received *Echinacea purpurea *extract and sham manipulation. The third group received placebo extract and true OMT. The last group received *Echinacea purpurea *extract and true OMT. Sequentially numbered, indistinguishable bottles of echinacea or placebo were provided in advance. After informed consent was obtained, the parent was given the next bottle in sequence, and the pharmacist was telephoned to determine which of two osteopathic physicians was assigned (also determined randomly). The osteopathic physician independently contacted the pharmacist to learn whether a child was to receive sham or true osteopathic manipulative treatment. Children, families, and pediatricians were blinded to the group assignment of each subject.

### Interventions

Children assigned to echinacea treatment received a 1:1 weight-to-volume 50% ethanol liquid extract of the fresh roots and dried mature seeds of *Echinacea purpurea *manufactured by Eclectic Institute, Inc. (Sandy, Oregon). All echinacea came from the same lot number, with an analysis by the manufacturer confirming 50% root and seed extract of *Echinacea purpurea*. A similar, identically labeled placebo prepared by Eclectic Institute contained 50% ethanol, 45% filtered water, food coloring and thickeners. Parents were instructed to give 0.5 ml orally 3 times daily for 3 days at the onset of cold symptoms, followed by 0.25 ml orally 3 times daily for 7 more days.

All children were scheduled for osteopathic sessions as soon as possible after entry, then 2, 4, 8, and 12 weeks later. At each visit, children assigned to OMT received diagnostic examinations and concurrent treatments as deemed necessary by the treating physician. Treatment modalities were limited to cranial osteopathy, balanced membranous/ligamentous tension, and/or myofascial release (applied directly or indirectly). These treatments consist of gentle manipulations of the cranium, pelvis, diaphragm, and other structures. No high velocity or thrusting maneuvers were performed. At the discretion of the osteopathic physician, an osteopathic percussion hammer could also be used for treatment, which allowed gentle vibration in tissues at variable frequencies. Children assigned to sham manipulation received an osteopathic examination only (palpation of the cranial bones and muscles and other structures) without treatment maneuvers.

The osteopathic physicians were well-respected members of the Tucson osteopathic community with practices restricted to manipulative treatment. All were members of the American Academy of Osteopathy and the Cranial Academy.

In addition to the study interventions, all parents received educational materials regarding otitis risk factors. If after enrollment a child received prophylactic antibiotics, otolaryngology referral, or tympanostomy tubes (usually for further recurrences of AOM), this information was recorded, but the child was allowed to remain in the study. Such decisions were made on clinical grounds by the treating clinician and were not influenced by the child's participation in the study.

### Follow-up

After enrollment, children were prospectively followed for 6 months, with monthly telephone contact, and availability for evaluation 5 days per week by study pediatricians (MBA and RAW) if AOM was suspected. Children diagnosed with ear infection during weekends were asked to see one of the study physicians early the following week to confirm the diagnosis. In addition, children received otoscopic and physical examinations at entry, at 3 months, and at 6 months by study pediatricians, who were blinded to the treatment assignment of subjects.

### Data collection

At enrollment, a questionnaire was used to collect information about the child's previous episodes of AOM, past medical history, cigarette smoke exposure, previous use of study alternative interventions, family history of allergy, and recurrent otitis media. The results of otoscopic and physical examinations were recorded at entry, 3, and 6 months, and at the time of any evaluation for possible AOM. At monthly telephone contacts, parents were asked if the child had any suspected or diagnosed ear problems during the previous month, and whether any side effects of the interventions were suspected. Parents were also asked at the 3- and 6-month visits whether they believed their child was receiving placebo for each study intervention.

### Main outcome measures

The primary study outcome was the occurrence of a first episode of AOM during the study period as defined above. The final analysis was performed using only episodes of AOM that were sufficiently well documented in the medical record to meet the definition as described in the paper. A secondary outcome was the number of episodes of AOM. For this secondary outcome measure, all physician-diagnosed episodes of AOM were included, without respect to the case definition for AOM.

### Analysis

Children were analyzed in the groups into which they were randomized. An initial analysis was done to determine if there was any important interaction between the effects of echinacea and OMT on the occurrence of AOM. Since none was found, the two interventions were considered independently. For each intervention, the cumulative incidence of AOM during follow-up was compared between treatment and placebo groups using chi-square analysis and the relative risk (RR) with 95% confidence interval (CI). P was considered significant at P < 0.05. The time to first episode of AOM was compared between groups using Kaplan-Meier analysis and the log rank test. Kaplan-Meier curves were truncated at 200 days of follow-up. Cox regression was used to estimate the relative risk of AOM during follow-up with adjustment for confounding effects of other factors. The median number of episodes of AOM during follow-up was compared using the Mann-Whitney nonparametric test. Analyses were performed using SPSS for Windows (Version 11.5.0, SPSS Inc., Chicago, IL) and EpiInfo (Version 5.01b, CDC and USD, Inc., Stone Mountain, GA).

### Sample size

We assumed the risk of having at least one episode of AOM during 6 months of observation among children with recurrent otitis media to be 60%. With no effect modification between echinacea and osteopathic treatment, a final sample of 100 children (50 subjects per group for each analysis), was needed to provide 80% power to detect a 50% reduction in the risk of AOM associated with the treatment being analyzed (alpha = 0.05). This magnitude of risk reduction is comparable to that observed with antibiotic prophylaxis [20].

The study was conducted with the approval of the University of Arizona Human Subjects Committee, and informed consent was obtained from a parent or guardian of each subject.

## Results

Ninety children were enrolled in the study, 74 from the university-based clinic and 16 from private practice sites. Subjects had a median age of 1.5 years. The majority was male (57%) and white non-Hispanic (44%) or Hispanic (39%). Despite randomization, there were notable differences among treatment groups in the frequency of potentially confounding factors, especially gender, presence of siblings in the home, frequency of AOM prior to study entry, and daycare attendance (Table 1).

**Table 1:** Subject characteristics according to assigned treatment group

	Randomized Treatment Group				
	Sham Manipulation		Real Manipulation		
	Placebo Echinacea	Real Echinacea	Placebo Echinacea	Real Echinacea	Total Sample
Characteristic	%	%	%	%	%
Age, years (median ± SD)	1.5 ± 0.9	1.5 ± 0.5	1.6 ± 0.5	1.6 ± 0.7	1.5 ± 0.7
Ethnicity					
White, non Hispanic	45%	41%	42%	50%	44%
Hispanic	41	41	38	36	39
Native American	9	14	4	-	7
Black, non Hispanic	5	5	4	9	6
Asian	-	-	8	5	3
Other	-	-	4	-	1
Male gender	68	59	50	50	57
Breastfed ≥ 6 mo.	27	23	29	32	28
Sibling(s) in home	64	68	54	50	59
AOM ≥ 5× in past year	32	64	30	23	37
Daycare attendance	41	64	58	64	57
Cigarette smoke exposure	23	18	25	14	20

Total number in group	N = 22	N = 22	N = 24	N = 22	N = 90

Six subjects (7%) withdrew or were lost to follow-up within 3 months of enrollment (1–2 in each group). Only 19% of subjects attended all 5 scheduled osteopathic visits, but 64% had 3 or more treatment visits (see CONSORT diagram [21]: Additional file 1). The percentage of subjects that came for follow-up examinations was 69% at 3 months and 62% at 6 months. Medical record information on the occurrence of AOM during at least 3 months of follow-up was available for 84 subjects (93%). The length of follow-up ranged from 2 to 278 days (median 183).

**Figure 1:**
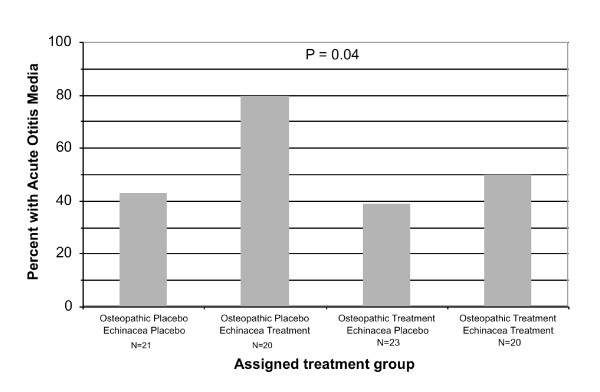
**Cumulative incidence of AOM according to assigned treatment group.** The P value is for at least one group being significantly different from the others.

Of 84 children followed for 3 months or more, 44 (52%) had one or more episodes of AOM as defined above. The cumulative incidence of AOM varied from 39% to 80% among the treatment groups (P = 0.04) (Figure 1). The highest rate of AOM was among children receiving echinacea alone.

The pattern of variation in incidence rates among the treatment groups did not suggest any plausible effect modification between echinacea treatment and OMT on the risk of AOM (Figure 1). In addition, a likelihood ratio test for interaction between the two treatments using Cox regression was not significant (P = 0.40). Therefore, the independent effects of echinacea and OMT were assessed separately.

**Figure 2:**
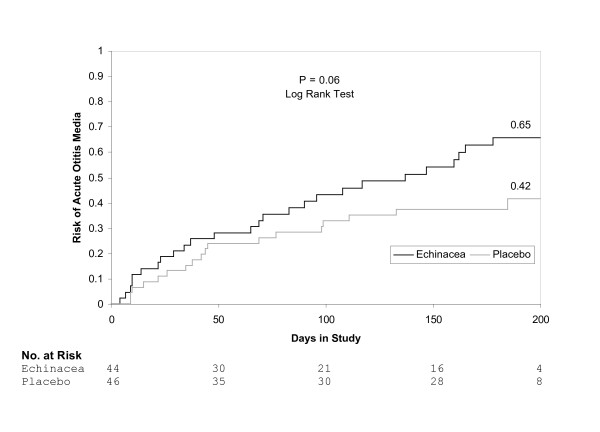
Kaplan-Meier estimates of the probability of AOM according to treatment with echinacea or placebo.

The use of echinacea was associated with an increased risk of AOM of borderline statistical significance. Sixty-five percent of children assigned to echinacea experienced AOM compared to 41% of children taking placebo (RR 1.59, 95% CI 1.04, 2.42). The Kaplan-Meier analysis using all 90 subjects yielded nearly identical results (Figure 2).

**Figure 3:**
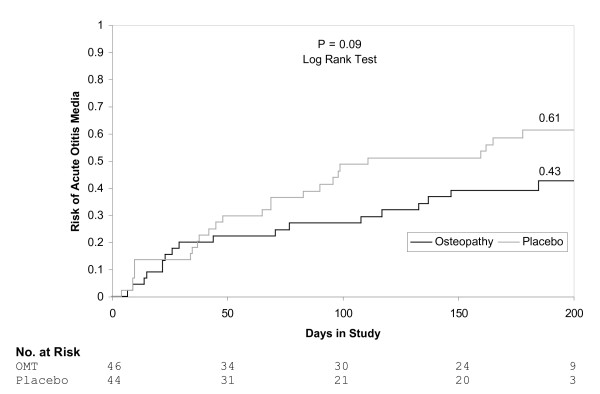
Kaplan-Meier estimates of the probability of AOM according to treatment with osteopathic manipulative treatment (OMT) or sham.

OMT was not significantly associated with the risk of AOM. Forty-four percent of children receiving OMT experienced AOM compared to 61% of children undergoing sham treatment (RR 0.72, 95% CI 0.48, 1.10). Restriction of the analysis to 56 children who had 3 or more osteopathic treatment visits did not change the findings (RR 0.70, 95% CI 0.41, 1.19). Results of Kaplan-Meier analysis using all 90 children were similar (Figure 3).

We estimated the effects of echinacea and OMT on the risk of AOM after adjustment for confounding factors using Cox regression in all 90 subjects (Table 2). The adjusted relative risk of AOM for echinacea treatment was 1.73 (95% CI 0.94, 3.18). The adjusted relative risk for OMT was 0.84 (95% CI 0.44, 1.61). Four variables (younger age, male gender, Hispanic ethnicity, and presence of one or more siblings at home) were independently associated with the risk of AOM and were included in the regression model.

**Table 2:** Cox regression estimates of the effect of echinacea and osteopathic manipulative treatment on the risk of AOM after adjustment for other factors

	Relative Risk	95% Confidence	
Variable		Interval	P-value
Echinacea treatment	1.73	0.94, 3.18	0.08
Osteopathic manipulative treatment	0.84	0.44, 1.61	0.60
Age (y)	0.49	0.26, 0.89	0.02
Male gender	2.15	1.09, 4.21	0.03
Hispanic ethnicity	2.02	1.10, 3.72	0.02
Siblings in home (1 or more vs. none)	1.96	0.99, 3.89	0.05

There was no significant difference in the median number of episodes of AOM during the study period between treatment and placebo groups for either echinacea or OMT. Comparisons were made using the Mann-Whitney nonparametric test.

One subject withdrew from the study following an adverse effect (vomiting after taking the echinacea placebo). One additional subject reported adverse effects (vomiting and non-urticarial rash two days after starting echinacea for a viral upper respiratory illness) but did not withdraw. Neither adverse effect was considered to have been caused by the study medication. As reported in monthly telephone interviews and at the 3- and 6-month visits, there was no statistically significant difference in reporting of any side effects between placebo and treatment groups for either echinacea or OMT.

**Figure 4:**
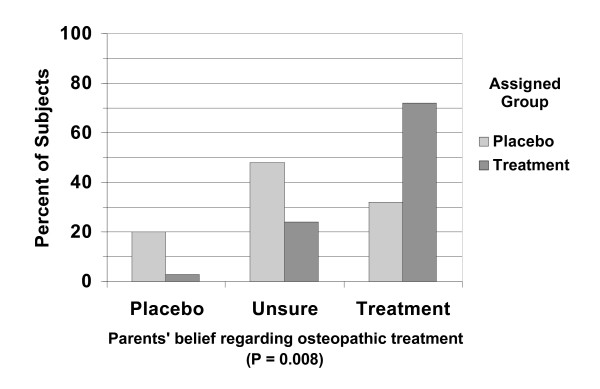
Parents' beliefs about osteopathic treatment assignment after 3 months in study (n = 54).

Parents were unable to distinguish whether their child was receiving echinacea treatment or placebo. However, when asked about OMT after 3 months, parents of children assigned to OMT were significantly more likely to believe their child was receiving actual OMT than parents of children assigned to sham (Figure 4). Even so, only 20% of parents of children assigned to sham treatments believed their child to be receiving placebo. Interestingly, the ability to distinguish OMT from sham treatment disappeared entirely by 6 months.

## Discussion

We found that preventive treatment with osteopathic manipulation did not result in a statistically significant decrease in the risk of AOM in otitis-prone children. Treatment with an alcohol extract of *Echinacea purpurea *root and seeds at times of upper respiratory infection was associated with a modestly increased risk of AOM. This effect was of borderline statistical significance and may have been due to chance.

As noted, the randomization process resulted in an unequal distribution of certain otitis risk factors among treatment groups. The group receiving echinacea alone appeared to be at higher risk based on several factors, and this chance occurrence may have been responsible for the higher rate of otitis in the echinacea group. We adjusted for known confounding factors, resulting in modest changes in relative risk estimates, but the possibility of incomplete adjustment, or unmeasured confounding by other factors cannot be excluded.

This is the first randomized, placebo-controlled trial of *Echinacea purpurea *for the prevention of AOM. We chose to treat children at the time of upper respiratory tract infections, rather than using a prolonged preventive regimen, based on the finding that echinacea appeared to be more effective in treatment than prevention of URI in adults [22]. Two subsequent randomized trials did not confirm this pattern in children. Taylor and colleagues found the pressed juice of *E. purpurea *aerial plant to be of no benefit to children in treatment of the common cold [16]. Cohen reported a significant reduction in colds and other respiratory outcomes following preventive use of a product containing liquid extracts of *E. purpurea *aerial plant, *E. angustifolia *root, propolis, and vitamin C [23]. The latter study also showed a decreased risk of acute otitis media with the echinacea product, but was not designed to measure this outcome in a rigorous way. Recent studies have confirmed our premise that AOM in children is a frequent complication of viral upper respiratory tract infection [24,25].

Research on the therapeutic use of echinacea is complicated by the fact that there are three species of echinacea in common use, and preparations are made from various combinations of leaves, flowers, roots, and seeds of the plant. Furthermore, various methods of preparation are used, including alcohol or glycerin extraction, desiccation, and juicing. These issues are discussed elsewhere with respect to the present study [26]. Our results cannot be generalized to other forms of echinacea.

One previous study evaluated OMT for prevention of AOM in otitis-prone children [7]. The authors reported a statistically significant decreased frequency of AOM episodes and fewer surgical episodes among treated children. That protocol called for 9 osteopathic treatment visits, compared with 5 in the present study. As noted by the authors, that study did not have a placebo control, and the control group had a very high dropout rate, a combination of factors with a significant potential to introduce bias.

The main limitations of this study were small sample size and incomplete compliance with osteopathic treatments and follow-up visits. Our final analysis involved 84 subjects, yielding 73% power to detect a 50% risk reduction under our initial assumptions. The borderline increased risk associated with echinacea treatment make a protective effect of this form and dosage schedule of echinacea very unlikely, despite the sample size.

Because we were able to identify episodes of AOM through medical record review, the incomplete compliance with follow-up pediatrician visits had limited impact on our findings. However, it is possible that the lack of a statistically significant benefit found for OMT was the result of inadequate compliance with OMT visits. An analysis restricted to subjects attending 3 or more treatment visits did not suggest a greater protective effect of OMT, but the sample size was small. On the other hand, research personnel exerted considerable effort at getting subjects to these visits, with limited success, perhaps because the children were not ill at those times. An analysis of the reasons for limited participation by families with OMT may be useful for future OMT research.

An ancillary purpose of this study was to determine if a suitable sham for osteopathic manipulation could be devised. A previous study of sham chiropractic treatment in children did not evaluate how parents perceived the sham treatment[27]. We hoped to be able to show that parents would be unable to distinguish examination alone from examination and concurrent treatment. In fact, about 20% of parents of sham recipients (versus 4% in the true treatment group) correctly thought that the child was receiving sham manipulation. However, the large majority of parents of sham recipients could not tell that the "sham manipulation" was placebo. Some osteopathic physicians believe any "hands-on" contact has potential therapeutic benefit. To the extent this is true, our "sham" treatment could mask an actual benefit of OMT.

## Conclusion

Treatment of upper respiratory infections in otitis-prone children with an alcohol extract of the roots and seeds of *Echinacea purpurea *does not decrease the risk of AOM, and may in fact increase risk. In the same population of children, a preventive regimen of from one to five osteopathic manipulative treatments over three months did not significantly decrease their risk of acute otitis media.

## Competing interests

The authors declare that they have no competing interests.

## Authors' contributions

Each author of this research paper has directly participated in planning, execution, or analysis of the study. RAW and MBA performed the clinical examinations on all study subjects. KAW coordinated the osteopathic assessments and treatments. KLG (now deceased) provided the subject randomization and assured that the other authors remained blinded to treatment arm protocols. MBA performed all statistical analyses. RAW, MBA, and KAW read and approved the final manuscript.

## Pre-publication history

The pre-publication history for this paper can be accessed here:



## Supplementary Material

Additional file 1 **CONSORT Wahl et al. CONSORT (Consolidated Standards of Reporting Trials) patient flow diagram.** Click here for file

## References

[B1] Casselbrant M, Mandel E, Rosenfeld R, Bluestone C (2003). Epidemiology. Evidence-Based Otitis Media.

[B2] Bluestone CD (1998). Role of surgery for otitis media in the era of resistant bacteria. Pediatr Infect Dis J.

[B3] Subcommittee on Otitis Media with Effusion, American Academy of Pediatrics (2004). Otitis media with effusion. Pediatrics.

[B4] Sawni A, Ragothaman R, Thomas RL, Mahajan P (2007). The use of complementary/alternative therapies among children attending an urban pediatric emergency department. Clin Pediatr.

[B5] Spigelblatt LS, Laine-Ammara G, Pless IB, Guyver A (1994). The use of alternative medicine by children. Pediatrics.

[B6] Shakeel M, Little SA, Bruce J, Ah-See KW (2007). Use of complementary and alternative medicine in pediatric otolaryngology patients attending a tertiary hospital in the UK. Int J Pediatr Otorhinolaryngol.

[B7] Mills MV, Henley CE, Barnes LL, Carreiro JE, Degenhardt BF (2003). The use of osteopathic manipulative treatment as adjuvant therapy in children with recurrent acute otitis media. Arch Pediatr Adolesc Med.

[B8] Galbreath WO (1929). Acute otitis media: including its postural and manipulative treatment. Journal of the American Osteopathic Association.

[B9] Frymann VM, Frymann VM (1998). Diagnosis and treatment of otitis media in children. The Collected Papers of Viola M Frymann, DO.

[B10] Pintal WJ, Kurtz ME (1989). An integrated osteopathic treatment approach in acute otitis media. Journal of the American Osteopathic Association.

[B11] Moresi A (1997). Otitis media: an Osteopathic approach. The Cranial Letter.

[B12] Roesler J, Emmendorffer A, Steinmuller C, Luettig B, Wagner H, Lohmann-Matthes ML (1991). Application of purified polysaccharides from cell cultures of the plant Echinacea purpurea to test subjects mediates activation of the phagocyte system. Int J Immunopharmacol.

[B13] Burger RA, Torres AR, Warren RP, Caldwell VD, Hughes BG (1997). Echinacea-induced cytokine production by human macrophages. Int J Immunopharmacol.

[B14] Luettig B, Steinmuller C, Gifford GE, Wagner H, Lohmann-Matthes ML (1989). Macrophage activation by the polysaccharide arabinogalactan isolated from plant cell cultures of Echinacea purpurea. J Natl Cancer Inst.

[B15] Melchart P, Walther E, Linde K, Brandmaier R, Lersch C (1998). Echinacea root extracts for the prevention of upper respiratory tract infections. Archives of Family Medicine.

[B16] Taylor JA, Weber W, Standish L, Quinn H, Goesling J, McGann M, Calabrese C (2003). Efficacy and safety of echinacea in treating upper respiratory tract infections in children: a randomized controlled trial. JAMA.

[B17] Barrett BP, Brown RL, Locken K, Maberry R, Bobula JA, D'Alessio D (2002). Treatment of the common cold with unrefined echinacea. A randomized, double-blind, placebo-controlled trial. Ann Intern Med.

[B18] Dowell SF, Marcy S, Phillips W, Gerber MA, Schwartz B (1998). Otitis media – principles of judicious use of antimicrobial agents. Pediatrics.

[B19] Subcommittee on Management of Acute Otitis Media, American Academy of Pediatrics (2004). Diagnosis and management of acute otitis media. Pediatrics.

[B20] Giebink GS (1994). Preventing otitis media. Ann Otol Rhinol Laryngol Suppl.

[B21] Moher D, Schulz KF, Altman DG (2001). The CONSORT statement: revised recommendations for improving the quality of reports of parallel group randomized trials. BMC Med Res Methodol.

[B22] Barrett B, Vohmann M, Calabrese C (1999). Echinacea for Upper Respiratory Infection: Evidence-based clinical review. JFamPrac.

[B23] Cohen HA, Varsano I, Kahan E, Sarrell EM, Uziel Y (2004). Effectiveness of an herbal preparation containing echinacea, propolis, and vitamin C in preventing respiratory tract infections in children: a randomized, double-blind, placebo-controlled, multicenter study. Arch Pediatr Adolesc Med.

[B24] Revai K, Dobbs LA, Nair S, Patel JA, Grady JJ, Chonmaitree T (2007). Incidence of acute otitis media and sinusitis complicating upper respiratory tract infection: the effect of age. Pediatrics.

[B25] Chonmaitree T, Revai K, Grady JJ, Clos A, Patel JA, Nair S, Fan J, Henrickson KJ (2008). Viral upper respiratory tract infection and otitis media complication in young children. Clin Infect Dis.

[B26] Mark JD, Grant KL, Barton LL (2001). The use of dietary supplements in pediatrics: a study of echinacea. Clinical Pediatrics.

[B27] Sawyer CE, Evans RL, Boline PD, Branson R, Spicer A (1999). A feasibility study of chiropractic spinal manipulation versus sham spinal manipulation for chronic otitis media with effusion in children. J Manipulative Physiol Ther.

