# Supplementary material for: Echinacea purpurea and osteopathic manipulative treatment in children with recurrent otitis media: a randomized controlled trial
Source: BMC Complement Altern Med. 2008 Oct 2;8:56. doi: 10.1186/1472-6882-8-56 (PMC2573879; doi:10.1186/1472-6882-8-56)

Echinacea purpurea and cranial osteopathic manipulative treatment in children with recurrent otitis media: a randomized controlled trial

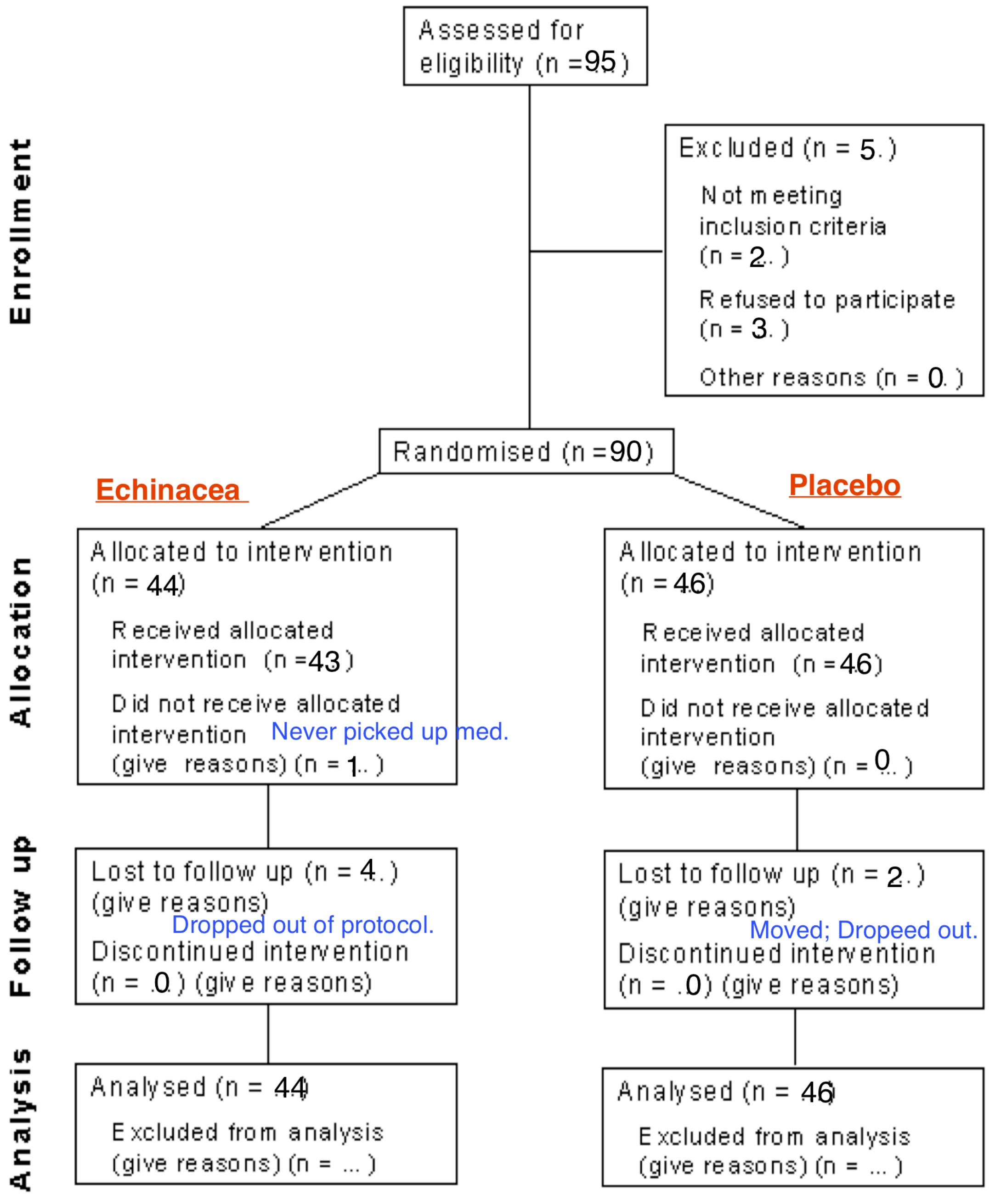

(Continued next page.)

***Echinacea purpurea* and cranial osteopathic manipulative treatment in children with recurrent otitis media: a randomized controlled trial**

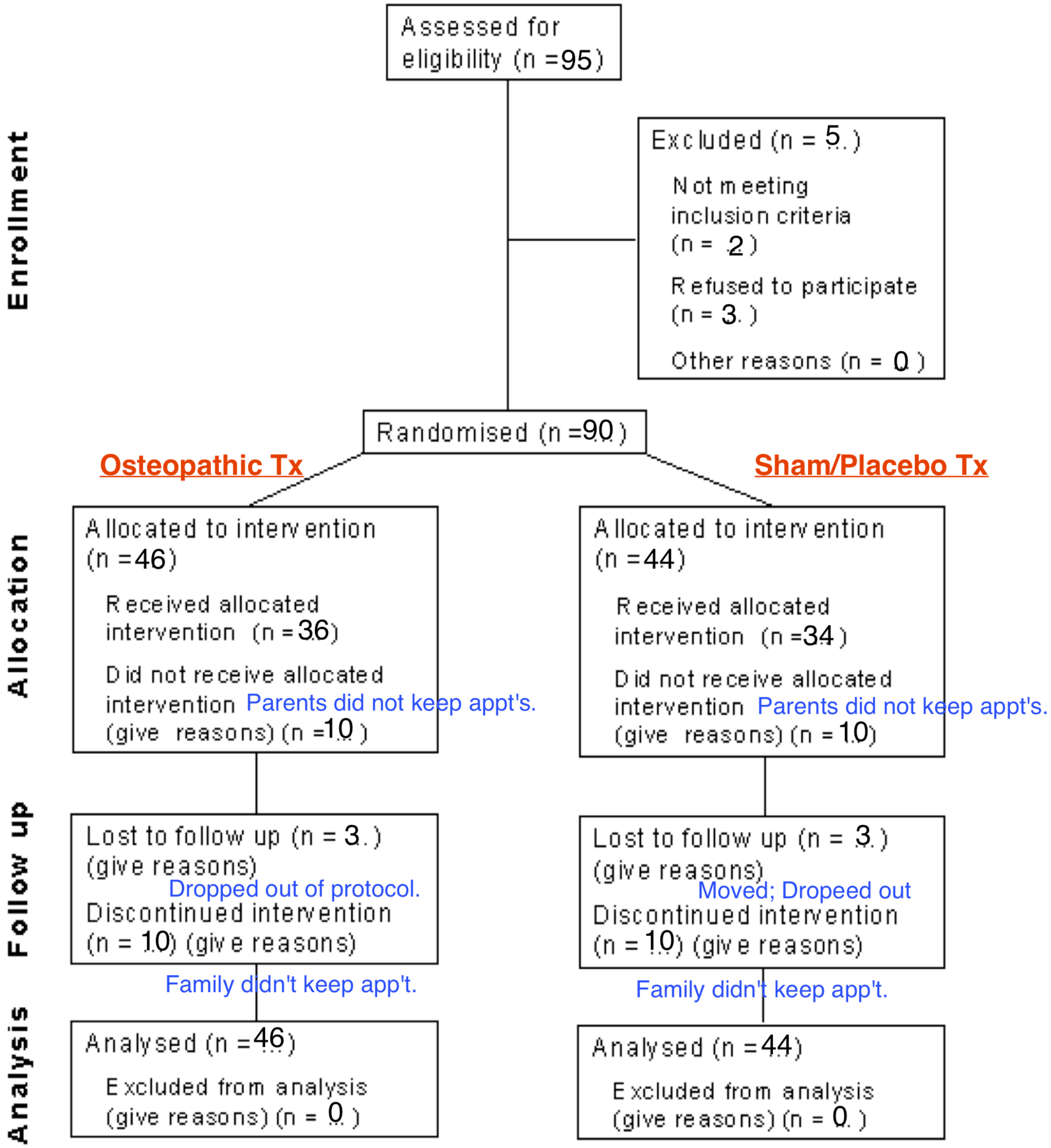

Supplement: Additional file 1 — CONSORT Wahl et al. CONSORT (Consolidated Standards of Reporting Trials) patient flow diagram. [file 1472-6882-8-56-S1.pdf]
